# Increased fairness in priority setting processes within the health sector: the case of Kapiri-Mposhi District, Zambia

**DOI:** 10.1186/1472-6963-14-75

**Published:** 2014-02-18

**Authors:** Joseph M Zulu, Charles Michelo, Carol Msoni, Anna-Karin Hurtig, Jens Byskov, Astrid Blystad

**Affiliations:** 1 Department of Community Medicine, School of Medicine, University of Zambia, P.O. Box 50110, Lusaka, Zambia; 2 Umeå International School of Public Health (UISPH), Umeå University, SE 90185 Umeå Sweden; 3 DBL-Centre for Health Research and Development, Faculty of Health and Medical Sciences, University of Copenhagen, Thorvaldsensvej 57 DK 1871 Frederiksberg, Denmark; 4 Department of Global Health and Primary Care, Kalfarveien 31, 5018 Bergen, Norway; 5 Centre for International Health (CIH), University of Bergen, Bergen, Norway

**Keywords:** Fairness, Priority setting, Health sector, Zambia

## Abstract

**Background:**

The challenge of priority setting (PS) in health care within contexts of severe resource limitations has continued to receive attention. Accountability for Reasonableness (AFR) has emerged as a useful framework to guide the implementation of PS processes. In 2006, the AFR approach to enhance legitimate and fair PS was introduced by researchers and decision makers within the health sector in the EU funded research project entitled ‘Response to Accountable priority setting for Trust in health systems’ (REACT). The project aimed to strengthen fairness and accountability in the PS processes of health systems at district level in Zambia, Tanzania and Kenya. This paper focuses on local perceptions and practices of fair PS (baseline study) as well as at the evolution of such perceptions and practices in PS following an AFR based intervention (evaluation study), carried out at district level in Kapiri-Mposhi District in Zambia.

**Methods:**

Data was collected using in depth interviews (IDIs), focus group discussions (FGDs) and review of documents from national to district level. The study population for this paper consisted of health related stakeholders employed in the district administration, in non-governmental organizations (NGO) and in health facilities.

**Results:**

During the baseline study, concepts of legitimacy and fairness in PS processes were found to be grounded in local values of equity and impartiality. Government and other organizational strategies strongly supported devolution of PS and decision making procedures. However, important gaps were identified in terms of experiences of stakeholder involvement and fairness in PS processes in practice. The evaluation study revealed that a transformation of the views and methods regarding fairness in PS processes was ongoing in the study district, which was partly attributed to the AFR based intervention.

**Conclusions:**

The study findings suggest that increased attention was given to fairness in PS processes at district level. The changes were linked to a number of simultaneous factors among them the concepts introduced by the present project with its emphasis on fairness and enhanced participation. A responsive leadership that was increasingly accountable to its operational staff and communities emerged as one of the key elements in driving the processes forward.

## Background

Because of the gross mismatch between the demand for health care and the availability of resources, priority setting (PS) is arguably one of the most important health policy issues of our time [1,2]. PS involves making a choice based on a ranking process, although occasionally the term is used as a synonym for rationing or resource allocation [3,4]. Priority setting entails formulating systematic rules in order to guide the distribution of limited health care resources among competing programs or categories of patients [5,6]. Well-known PS models have been grounded in theories focusing on social justice, equitable allocation, efficiency and burden of disease. However, there is commonly disagreement about which values should dominate PS philosophy [7]. In the absence of consensus, there has been a move away from a search of basic principles associated with the desired health outcomes and their distribution. There has, in recent years, instead been increasing emphasis on systems of PS that can ensure stakeholder and user involvement [8]. A new focus on legitimate and fair PS has emerged [9] to ensure fundamental values such as trust in the decisions made [10,11].

The AFR framework was developed by Daniels and Sabin [12], and has received growing consideration in recent years. AFR is founded in justice theories emphasizing democratic deliberation, and is focused on the actual priority setting process. The main idea behind the framework is that while consensus on distributive principles is not to be expected, what should be aimed at is agreement on a process that produces decisions which are perceived as being legitimate and fair by the stakeholders [13]. A focus on process can promote a relatively consistent treatment of similar cases, or so-called ‘formal fairness’. The AFR approach to PS has primarily been developed and tested for applications in health care organizations and within fairly well-defined settings such as health institutions in high income settings [14]. The present research endeavour represents an attempt to apply the AFR framework at district level in low income context.

According to Daniels and Sabin, legitimacy and fairness need to be located at the heart of PS processes. Legitimacy involves questioning why and under what conditions authority over PS should be placed in the hands of a particular organization, group or person, whereas fairness relates to questioning when users and providers of services (a patient or clinician) should accept a particular PS ruling as fair. According to AFR, a fair PS system has to meet the following four conditions: *relevance, publicity, appeals and enforcement* (Table 1) [14,15]. These conditions have over the previous few years been applied more frequently to guide operations to enhance fair and legitimate PS processes [11-15].

**Table 1:** **Definitions of the four conditions of AFR framework [**[14]**,**[15]**]**

**AFR condition**	**Meaning of AFR condition**
1. Relevance condition	Decisions should be made on the basis of reasons which appeal to evidence, principles, and arguments that ‘fair-minded’ people can agree are relevant under the circumstances.
2. Publicity condition	Decisions and their rationales must be publicly accessible so as to stimulate public debate on PS.
3. Appeal & revision condition	There should be a mechanism for challenge and dispute resolution regarding limit setting decisions, and more broadly, opportunities for revision and improvement of priorities in the light of new evidence or argument.
4. Enforcement condition	There should be either voluntary or public regulation of the process to ensure that the above three conditions are met.

The AFR framework aims to provide a guide to enhance the likelihood of an acceptable outcome in PS matters. If limit setting priority setting decisions are based on evidence, reasons and principles perceived as relevant among stakeholders, made more publically available and capable of being challenged, it is presumed that they will become more appropriate and acceptable in a local context. Their results or implications are consequently expected to become more sustainable.

In 2006, the AFR approach to enhance legitimate and fair PS was introduced by researchers and decision makers within the health sector in the EU funded research project entitled ‘Response to Accountable priority setting for Trust in health systems’ (REACT). The project aimed to strengthen fairness and accountability in the PS processes of health systems as well as to assess the influence of the processes on indicators of quality, equity and trust in the service provision as means to achieving sustainable health improvement at district level in Kapiri-Mposhi District, Zambia, in Mbarali District, Tanzania and in Malindi District, Kenya [10]. This paper focuses on local perceptions and practices related to what was perceived as ‘fair’ priority setting (baseline study) and the potential evolvement of such perceptions and practices over time as a result of an AFR based intervention (evaluation study) in Kapiri-Mposhi District, Zambia.

## Methods

### The study setting and PS structure

Kapiri-Mposhi district is located in Central Province in Zambia. The district had a population of 240,841 in 2010 [16]. The district has one hospital, four health centers and 22 health posts. The district is demarcated in four main zones, each zone having one health center.

**Figure 1:**
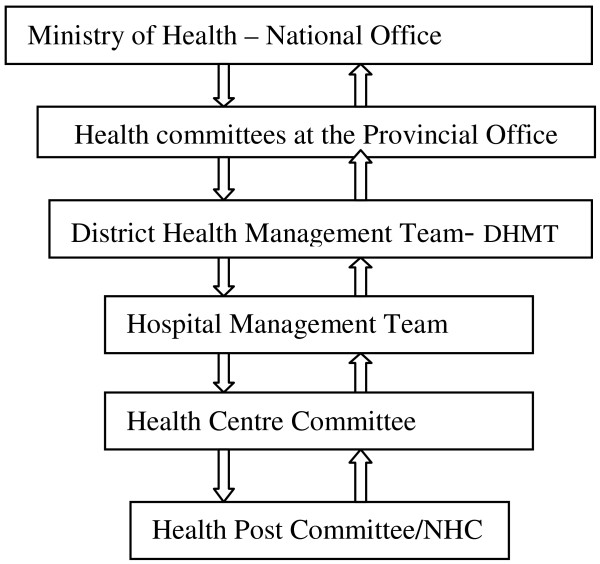
Simplified structure of the PS process in the health sector in Zambia.

In terms of its position in the structure of priority setting (PS), Kapiri-Mposhi like other districts of Zambia is located at the third level in the health-related hierarchy (Figure 1). The top level is the Ministry of Health (MoH), i.e. the national office. It is at the MoH that the planning guidelines and budget estimates are developed. The second level is the Provincial Office (PO). The role of the PO is to coordinate the health services within the province, and as such acts as a link between the MoH and the District Health Management Team (DHMT).

### The health related PS process in Zambia

Renewed attention given to the challenging planning and PS processes in health began in the early 1990s in the country when the MoH adopted a decentralized management model of the health services as well as a set of financing reforms. The guiding pillars in the implementation of the health reform process included quality leadership, accountability and partnerships at all levels. The annual planning cycle starts with the Provincial Health Director (PHD) and other provincial officials attending the national planning launch at the MoH’s national offices, where guidelines and central issues for consideration in the following year’s budgets are presented and discussed (Figure 1). The Provincial Health Director is presented with indicative figures from the MoH which each district within the province is expected to follow when setting their priorities. For the period 2006 to 2011, the PS process for health in Zambia was driven by the National Health Strategic Plan 2006-2011 (NHSP) developed by the MoH. The National Health Strategic Plan is part of and feeds into the Fifth National Development Plan (FNDP) for Zambia which in turn is led by the Medium Term Expenditure Framework (MTEF).

Once the guidelines and estimates have been communicated from the province to the districts, the District Health Management Team (DHMT) undertakes an evaluation and invites the teams from the first level hospitals and health centre teams (those in charge and others) to a meeting. At the meeting, they discuss the guidelines and the respective health service and financial data around which to make their plans and budgets. These teams then proceed to engage the various health centre/health post committees as well as community structures such as the neighbourhood health committees (NHCs). The plans/budgets are submitted to the DHMT which eventually consolidates them into a joint district plan. This plan is subsequently forwarded to the province and finally to the national office. This system is spelled out in Figure 1. The process engages community structures (e.g. NHCs) and was termed ‘bottom-up approach’ for PS of primary health care service (PHCS) provision [17-19].

### The REACT project in Kapiri-Mposhi District

A preliminary phase in the implementation of the AFR framework in Kapiri Mposhi district began in 2006, involving the collecting of baseline data, consultation and planning. The full introduction of the AFR concepts and principles was initiated in 2008, a continuous assessment followed for a period of two years with a final assessment of the potential change in the manner the AFR conditions were guiding the application of the AFR framework in PS processes. This final phase took place in 2010 [10,20].

The introduction of the AFR conditions and the subsequent follow up in Kapiri-Mposhi was carried out by the DHMT with support from an Action Research Team (ART). The Action Research Team comprised four researchers from the University of Zambia, three core members from the DHMT and a focal point person (social scientist resident in the area). The role of the ART was to facilitate and assess or monitor opinions and practices based on the AFR criteria (or lack of such) through participant observation during DHMT meetings, through observations at health facilities and through appropriate documentary reviews at district level.

The District Medical Officer (DMO) was fully engaged in the project from the onset, and worked throughout the project period to introduce and adopt the AFR conditions into the daily routines at district level. The DHMT was supposed to facilitate the application of the AFR conditions during the annual planning meetings, during stakeholder meetings, and in the day-to-day decision making processes that concerned choices between diverse options in a context with severe resource limits. The ART attended six DHMT meetings for observation in 2009 and 2010. AFR related project meetings were moreover convened annually during the project period. Three other stakeholders meetings were also conducted for assessment and follow up. This latter category of meetings consisted of meetings with the ART team including members from NGOs and neighbourhood health committees (NHCs).

The ART adopted a Describe-Evaluate-Improve (DEI) approach in documenting the process during the meetings. The focus of these meetings was thus to describe, evaluate and improve the incorporation and use of the four AFR principles in assisting the continuous PS processes. The description component implied an assessment of how decision makers and PS groups related to the four conditions .i.e. how the health centres in charges, health management teams and boards, district administration, etc. related to them or observed them when setting priorities. The evaluation element inferred an assessment of progress or lack of progress (identifying gaps) in terms of applying the conditions. The improvement part estimated potential modifications or advances in the employment of the conditions, exploring examples or lack of examples of such modifications or examples of the planning and implementation of prospective changes in attempts to revisit or ‘repair’ cases where the conditions had not been properly drawn upon in the PS process.

### Recruitment of informants

The participants were purposely recruited based on their role in PS processes. In both the baseline and the follow up studies, the participants included persons who played key roles in PS at the provincial office, DHMT, hospital, health centre and health post levels. Other partakers were recruited from the NGOs and government departments that worked closely with the MoH in PS.

### Data collection techniques

Data collection involved in depth interviews (IDIs), focus group discussions (FGDs), as well as documentary review of relevant documents related to health management and PS in Zambia, as well as minutes and observations from ART meetings. In the baseline study, 49 in depth interviews (IDIs) and five focus group discussions (FGDs) were conducted (Additional file 1). During the evaluation phase 18 IDIs were completed (Additional file 2).

The main sources of the evidence for both the baseline and evaluation phases were the IDIs. In the baseline phase, evidence from IDIs was triangulated with data from the FGDs and review of documentation of PS and other decision making processes. For the evaluation phase, evidence from the IDIs was triangulated with data from the observations, minutes of the ART meetings and other documents. Triangulation can take different forms as discussed by Patton [21]. The first approach involved methods triangulation, which meant assessing the consistency of findings by comparing data patterns across the material generated by different methods, i.e. between the FGD and the IDIs in the base line study and between the IDIs and the minutes from the AFR meetings during the evaluation study. The second type of triangulation involved searching for potential patterns in terms of continuity or change in the collected material by scrutinising the data collected with the same method but at different points in time during the data-collection period. Furthermore ‘analysis triangulation’, implying a process where multiple researchers are engaged in the analysis of the findings (i.e. all authors of the study), was also employed. The patterns and potential variations that in particular were searched for during the analysis phase were indications of the level of stakeholder participation in decision-making processes, the use of diverse communication and appeal processes, indications of the use of official guidelines for resource allocation and the emergence of local concepts of fairness.

Trained social scientists from the University of Zambia (who were part of REACT) conducted both the IDIs and FGDs. Semi-structured interview guides were employed to guide the IDIs, whereas topic guides were used to guide the discussions during the FGDs. Both tools were administered in English, as all the study participants spoke English well. The questions were structured around the manner in which central AFR concepts (fairness, accountability, the four AFR conditions), were incorporated in the PS process. Other major themes covered in the interviews were general aspects of decision making such as the processes of setting priorities and criteria used in PS. The interviews taking place in the evaluation phase also included questions considering conceivable changes in PS processes with particular reference to the AFR based and other related interventions.

### Data analysis

All interviews were recorded digitally and later transcribed verbatim. Data was transcribed by the first and third paper authors. Analysis during both the baseline and the evaluation phases started while in the field, with a later thorough reading of all the interview transcripts in order to get to know the overall data set well. The study adopted a thematic structure analysis which involved rigorous identification of relevant codes pertaining to the content of each segment of the interviews, and the subsequent classification of the material according to emerging patterns of major topics or themes [22]. The focus was placed simultaneously on identifying recurring content or patterns in the material as well as identifying nuances or new emerging themes of relevance for priority setting and decision-making processes. During the coding process substantial emphasis was placed on retaining the original meaning of what was being communicated by the informants.

The analysis process took place in two separate processes. The first was carried out with the use of NVIVO version 7 (QSR Australia) by the first and third authors. The analysed material was later revised during a workshop which was attended by all members of the REACT team from the University of Zambia and the District Medical Officer for Kapriri Mposhi district. Both coding processes involved the matching of codes (i.e. one- or two-word statements summing up the content of particular sentences or paragraphs) with segments of text/informant statements selected as representative of the code. The workshop provided an opportunity to review and revise the initial codes, in a process that scrutinised the credibility of the codes by returning to the transcripts (some new codes were identified at this point).

A code manual was then developed based on the identified codes. The code manual was developed with the key questions and the theoretical underpinnings provided by the AFR framework in mind. This assisted the process of identifying the larger themes in the material. This part of the process included the coordinating of the codes along lines of common major themes/topics.

Codes with similar meanings were linked/matched in larger themes. This was an iterative process which involved the re-reading of codes (and sometimes transcripts and other raw data) and relating them to the themes. It included moving back and forth between the codes/topics/themes and the data sets multiple times as described by Fereday et al. [23]. For example, codes such as *treating different people in the same manner* and *absence of segregation* were eventually categorised under the theme *equality/ impartiality,* and the code *stakeholder participation* was eventually placed under the theme *legitimacy*. The final stage consisted of corroborating and legitimating the themes which involved closely scrutinising the previous stages to ensure that the clustered themes were representative of the initial data analysis and assigned codes.

Data from the IDIs, the FGDs, and reports of the AFR team’s observations and minutes were triangulated to assess similarities and differences. This involved a new review to ensure the integration and synthesis of data from the different data collection sources through examining and comparing the different data sets with the aim of exploring the material’s validity. Although, the analysis is presented like a linear process, it should be emphasized that it involved continuous shifting back and forth between the different data sets as well as between the participants’ narratives and the researchers’ interpretation of the meanings of the material [24].

### Ethics

The University of Zambia Research Ethics Committee approved the protocol for this study in 2006 (IRB number 1131, approval certificate FWA 338). Oral informed consent was sought from all study participants and anonymity was assured throughout the study. No pressure was asserted in the recruitment or in the interview process. Informants’ confidentiality and anonymity was secured throughout the study.

## Results

This results section describes perceptions, concepts and practices related to priority setting (PS) processes as they emerged over a period of three years (2008-2010) among our study participants. It presents an assessment of a process in which REACT’s Action Research Team (ART) and other stakeholders were involved in an attempt to enhance fair PS. Despite the process focus, we do for the sake of organizing and overview, present the findings through a baseline study (a ‘before’ phase) and an evaluation phase (an ‘after’ phase), and with reference to the assessed status and changes in each AFR condition (before and after) the AFR based intervention.

The baseline study revealed that fairness-related concepts were commonly employed in Bemba (the local language), and institutions and practices were in place that were perceived to enhance fairness in priority setting. During the evaluation phase of the study, there were indications of favourable changes that had taken place during the three years, changes in which even more emphasis was placed on fairness and inclusiveness. Inclusiveness is an expression of a representative stakeholder participation in decision making. Inclusiveness is one of the criteria for legitimacy of decisions. The relation to existing plans and the involvement of other providers and of the users ensures inclusiveness. The apparent changes emerged at a conceptual level, but also in terms of indications of shifts towards somewhat more inclusive PS processes at the level of actual practice. The interviews taking place at the evaluation stage moreover demonstrated increased awareness and use of AFR notions. Other relevant processes of transformation were taking place at the time as the project intervention pulling in the same general direction, making it impossible to link potential change directly or solely to the AFR process.

### Defining the AFR ‘relevance’ condition through equality and impartiality concepts - the baseline study

According to the relevance condition of AFR, rationales for priority-setting decisions should aim to provide reasonable explanation of why they were taken. More specifically, for a rationale to be perceived as reasonable, it should be based on evidence, values, reasons or principles that are accepted as relevant by the stakeholders within the given sector, institution or locality.

Analysis of the IDIs and FGDs showed that there were common terms, principles, phrases as well as practices that implied fairness, or indicated fairness in a PS context well in place before the introduction of the AFR intervention. Central Bemba terms or principles implying fairness included *‘ulinganya’* and *‘ukushikwete akapatulula’. ‘Ulinganya’* means treating different people in the same manner or equivalently. Employed in a PS context, the term means applying measures, rules or guidelines equally when handling diverse issues. The expression ‘*ukushikwete akapatulula’* literally means absence of segregation, i.e. impartiality or being just in handling diverse issues. One of the FGD participants explained the concept of *ulinganya as* follows:

*“In our language…fairness is ulinganya’ literally meaning* equality. *This means everyone should get an equal share of the cake.” (Staff, DHMT).*

According to the responses given during the IDIs and FGDs, both concepts (*ulinganya* and *ukushikwete akapatulula*) were commonly applied in planning processes. Equality and impartiality principles were regularly utilized during human resources planning sessions, especially deployment of resources for health. There are, for example, human resources committees within the DHMT which attempt to ensure that fairness prevails in processes of employing new staff. These committees follow standardized guidelines which stipulate that staff should be deployed to various health facilities in accordance with the size of the health facility and the services offered, i.e. in accordance with overviews of available staff and infrastructure.

To ensure that the PS is perceived as fair, informants held that the district accommodated the views of a large number of and various categories of organizations and community members. Informants held that stakeholder participation in PS is important as it improves the likelihood that the decisions reached are perceived as appropriate for the particular context. Inclusion of various segments of the population also enhances the experienced legitimacy and ownership of the final resolutions, and consequently the participation of stakeholders in the implementation and monitoring of the decisions. In addition, legitimacy helps in understanding why and under what conditions authority over PS is placed in the hands of particular stakeholders.

Detailed analysis of the IDIs revealed that the district attempted to apply a participatory approach in setting priorities during weekly, monthly and annual planning meetings. These meetings were generally said to be conducted in a democratic and open manner where effort was made to ensure that no one person dominated. Some of the local concepts or phrases used to describe stakeholder participation in these meetings were ‘*ukuibimbamo’* meaning, being part of a process*, ‘kulanshanya nama partners’* meaning consulting key stakeholders, and *‘ukupandana amano’* meaning sharing knowledge. As one DHMT member expressed it in an IDI:

*“We do hold weekly meetings to evaluate activities and set new plans…I think that the meetings are fair because….if there is a problem we find the solution together.” (Staff, DHMT).* The Staff at the DHMT added that *“everyone is free to talk about the problems the department is facing.” (In Bemba: ‘bonse balandapo*; *cilamuntu alandapo’).*

The FGDs in particular indicated that before the introduction of AFR, the district employed guidelines developed by the MoH which recommended bottom up approaches in PS processes. Each district team is in such processes expected to strategize along the stipulated lines, and along corresponding indicative planning figures. At the district level, the planning process was said to start with the Neighbourhood Health Committees (NHCs) located at the health centers in the various zones. These committees identify and prioritize among their experienced needs. The lists of priorities are submitted to the DHMT for assessment and consolidation into the district’s annual plan. This process of consolidating diverse health centre reports was undertaken by the nurses in charge of rural health centers and by the heads of departments at the district offices, though with direct involvement or feedback for committee members in this part of the process. The principle of engaging committees in PS was described as ‘*Kubombelapamo na kabungwe’* meaning ‘working together with the committees’. It was noted that the district adopted the principle of local inclusion in PS processes as early as 2002, and that it was a central motto for the DHMT.

Other informants emphasized that the views of the stakeholders in the community were also taken into account, referring to the district’s motto.

“First we consult other stakeholders in the community before coming up with plans. This is actually part of the motto for the DHMT which is: ‘Providing quality health services in partnership with the community’ (In Bemba: ‘ukuleta imilimo isuma iya ubuumi mukubombela pamo nabena cipanda’). (Staff, DHMT).

Despite the concepts and organisational structures being in place to ensure involvement from the community level and up to the district level, many of the study participants argued that the shortage of funds would often hinder the DMHT in addressing their agreed priorities and involved a necessary re-priority setting or resulted in more ad hoc decision making, which did not base on the procedural guidance for involvement beyond the core executive team of the DHMT. As shortage of funds for actually approved budgeted activities was common, a major part of actual priority setting was not adequately reaching other involved organizations and even less the communities in order to get the views and priorities of ordinary people when planning for provision of services. One FGD participant explained that the challenge from both the limited involvement and feedback from the formal priority setting decisions as well as changes to them was due to a number of uncontrolled reasons during the year:

“But where I can bemoan is on the grassroots, the grassroots have sort of been marginalized. Because you find that…decisions are made by the technical team, i.e. going upward, but the grassroots are left out…” (Staff, DHMT).

### Modification of practices in line with the AFR ‘relevance’ condition - the evaluation study

During the evaluation phase of the study, informants held that there had been an increased awareness of the importance of making stronger efforts to ensure the application of equality and impartiality principles in PS processes. At the time of the periodic observations and the monitoring of the PS processes by the ART as well as during the review of the ART minutes, increased awareness of the ideas linked to fair PS processes as perceived from an AFR point of view were documented. In addition to continuous reference to the concepts and organisational principles and institutions brought out during the baseline study - such as equality and impartiality concepts and structures of inclusion - most study participants would add features such as ‘transparency’, ‘accountability’ and ‘equity’ as key components of fairness and fair PS processes. Transparency and accountability were said to imply that resources were used in accordance with recognized principles and procedures, usage of resources was open for review and that reports revealing what funds have been used for are submitted in an appropriate and timely manner.

It was also noted that a greater emphasis on the principle of ‘equity’ had emerged, e.g. through the ways in which the DHMT tried to prioritize and provide resources for the most vulnerable people in the community. One IDI informant provided an example of how this surfaced in practical politics: *“There have been improvements in ensuring that there is equity in the distribution of resources. For example, there are ITNs (mosquito nets) that come for under five and pregnant women. But you see, because one might be a wife to the chief - though she may not be pregnant but would nonetheless want to get a share. (Then) I say no. I say let’s take the resources where they are supposed to be taken.” (Staff, Health Center).*

In an attempt to strengthen the legitimacy and potentially contribute towards improved relevance of PS, many of the informants held that decision making processes had been broadened to include staff within the health sector that were not part of the top management, and who had earlier not been consulted making the processes more inclusive. It was argued that there was an increasing recognition of the need for finding ways of engaging more participants from the grassroots level, as well as paying special attention to the needs of particularly vulnerable segments of the population. Particularly, the analysis of the IDIs and the minutes of the planning meetings at the district revealed that informants held that there had also been an enhancement of accommodation of the views of diverse organizations, e.g. non-governmental organizations active in the district such as Corridors of Hope, SKOWA, Family Health Trust, CARE International, etc. This last category of stakeholders had earlier not been involved in health related PS and decision making processes at district level in Kapiri-Mposhi. New governmental institutions at district level, such as the Ministry of Agriculture Food and Fisheries, were also expressed to be more likely to be asked for input and advice. It was said that getting the views of such a large variety of parties helped achieve a greater overview over the diverse perspectives and priorities existing within the district.

The management had also tried to broaden the participation through strengthening committees at DHMT level which had become almost non- functional. According to the documents on operational committees at the district level, these included the finance, human quality assurance, infection and prevention, tender and procurement and disciplinary committees. Both IDIs and observations of meetings moreover showed that membership in these committees was increased by including staff that had not commonly been a part of the DHMT.

“The number has grown…there are people from DHMT, also the hospital staff attend, the nursing sisters from health centers, representatives from neighborhood health committees, we are quite many this time. For a long time what used to happen was that just a few officers would attend and then they would come to tell us whatever was discussed”. (Staff, DHMT).

The DHMT moreover tried to enhance community involvement in PS processes by encouraging regular meetings between the newly established committees and the community structures. An example of heightened community contribution in PS processes in health that was mentioned was related to the introduction of AFR practice satellites for the Action Research Teams (ART) at the health centers in the four main zones*.*

### The AFR ‘publicity’ condition - the baseline study

A central condition for ensuring fairness according to the AFR framework is by making sure that priorities and their rationales are made publicly accessible to the various categories of stakeholders. During the baseline study most informants did not immediately cite local publicity related concepts when defining fair PS. In terms of actual practice, it was reported that the district had restricted ways of communicating decisions to stakeholders, which according to the DHMT was primarily due to limited funds. However, several other reasons from the involvement constraints - illustrated under the relevance condition - were also cited as barriers to communication. The review of the district reports confirmed that approaches to the fruitful communication of priorities were still not properly developed.

### Increased visibility of the AFR ‘publicity’ concept - the evaluation study

During the evaluation phase of the research, the study participants recorded improved awareness in terms of defining publicity as an aspect of fairness and fair PS processes. It was stated that although the economic limitations continued to place severe restrictions on attempts to more inclusive involvement, the district made serious attempts at strengthening ways of publicizing priorities. The review of documentation on communication systems at district level as well as observations of communication patterns showed that information for PS was progressively communicated through memos, notices, megaphones and sometimes through door to door campaigns with the use of the neighborhood health committees. In certain cases, it was conveyed through churches, schools, distribution of posters and committee meetings. In order to reduce the costs for meetings and to encourage more local stakeholder involvement, the district preferred holding more meetings at local level as opposed to having them at DHMT level. Such information dissemination efforts were said to have increased.

Despite putting in place such measures, publicity was still said to be too limited due to the limited economic resources, low literacy levels and lack of interest by many local people in expressing their views during formal or informal opportunities for participating in PS processes. Many felt that participating in PS processes in health was only relevant for staff working within that sector.

### The AFR condition ‘appeal and revision’ - the baseline study

Fair PS does not only imply the proper inclusion of many stakeholders when developing priorities, but also involves a system for ensuring the possibility of revising decisions after an intervention/priority has been imposed. It was however noted that challenging or appealing against the decisions that they deemed were not fair was very difficult and was not perceived to be an option by most informants, even at higher levels in the administration chain.

Informants moreover held that there were no clearly spelt out ways through which the community could channel their views whether in support of or against the priorities set at higher levels. The demand to follow standard recommendations coming from higher levels was in fact perceived by many as incompatible with a participatory PS approach. They said that the district would often have specific needs which did not necessarily fit within the priorities outlined in the government guidelines, but that these had to always be followed.

### Increased emphasis on the ‘appeal and revision’ condition of AFR - the evaluation study

Informants reported that they had also noticed changes in relation to appealing against certain decisions over the three years period. It was suggested that in the last couple of years, an increasing openness towards appeal processes had at least been initiated, albeit not at a large scale. It was held that earlier all appeals at the community or health facility level had to be channelled through the DHMT, but that over the past few years, appeal procedures had been established at health facility level. The review of documents found, for example, that at one health facility some cases were being handled at health facility level. This possibility was deemed fundamentally fair as it provided an opportunity for people also at the lower level to air their views when they were not happy with a decision. On informant stated:

“*In the olden days, once management made a decision it was final, unlike now. If you make a decision and the decision is not favored by the people, and they come and they try to appeal, then you revisit (the issue) or probably change it. This has been a result of REACT.” (DHMT member).*

However, even though some informants acknowledged that the opportunities for appeals had been enhanced, most stated that actual changes of decisions that would have financial implications were in practice not possible to make due to the severe budget limitations. It has earlier been expressed how the actual allocations frequently get delayed or fall short of the budget and that involvement including appeals for decisions should also in such situations include communities in the re-prioritization.

### The role of the AFR condition ‘leadership’ in promoting fair priority setting - the baseline study

The AFR approach to PS further requires having techniques for regulating the decision making process to ensure that the relevance, the publicity and the appeal-conditions are met. Informants in both FGDs and IDIs readily acknowledged that leadership skills are relevant for promoting fair PS. They unanimously argued that the present leadership in the district tried to aid broad stakeholder involvement and upheld equality and equity principles in PS processes and efforts.

### The AFR conditions ‘leadership and enforcement’ key to improving fair PS - the evaluation study

Several informants held that the present good leadership skills in the district helped in promoting AFR conditions in the employment of fair PS processes. The leaders in the district did not change from the time of the baseline study to the point of the evaluation study which ensured continuity and commitment in the administration. The study informants reported that the leadership actively continued to promote broad and inclusive participation in decision making processes and in expanding awareness about AFR related principles.

“Apart from REACT, I think that good leadership skills and attributes in the district have contributed to improved fair PS processes.” (Member of the Provincial Health Team).

Improvement in terms of transparency and accountability was reported as being a part of the larger commitment at district level towards enhanced openness and inclusion. The improvement could be detected at various levels. For example, it was deduced from the audit report for the year 2010, that compared to the previous year, it had fewer audit queries.

*“When the audit report was produced…the auditors said that in this year’s audit report there were no major queries.” (DMO).* The DMO felt that this was surprising but linked it to the increased focus on accountability in the district.

The mounting commitment from the existing government towards improvements in accountability and transparency principles at every level was partly attributed to the limited funding going into the health sector; with extreme economic constraints, the ways in which the funds were spent had to be open and clear and had to be accounted for. *“What has changed, as I said, is that we are able now to sit down as a team and make a decision together at every level which was not the case before. Maybe because we had a lot of money, there were so many donors in the MoH. But now things have changed, we don’t have enough resources…and then at this point people have understood that there must be stewardship, accountability, and transparency…We have seen that the concepts that we have learnt are helping us.” (DMO).*

Among examples of the influence of increased attention to involvement, fairness and equity, the DHMT further attempted to within available resources optimize equality of service provision, communication and publicity through more efficient allocation of the two vehicles in the district. Observation showed that one of the vehicles was kept at the district headquarters while the other was given to a rural zone for more easy access to the communities. Partnering with the Churches Health Association of Zambia, the DHMT had moreover managed to arrange a vehicle for one of the other rural zones. The extreme scarcity of funds thus seemed to help strengthen the PS process to move in a direction where the activities perceived to be the most critical were given priority.

## Discussion

The study revealed that concepts and ideals of fairness in priority setting (PS) pertaining to equal share and inclusiveness were well established prior to the coming of REACT. Views and practices such as equality, impartiality, stakeholder involvement, bottom up approaches and partnerships with community structures had been emphasized as central in discussions of fair PS for a number of years. The principle of strong partnerships with the grassroots was not only a part of the local perceptions of fair PS but was enshrined in the motto of the district.

The detailed minutes of the Action Research Team (ART) proceedings, the observations taking place throughout the project period, as well as the interviews during the evaluation study nonetheless suggest that transformation of both concepts and practices had taken place, not the least in the DHMT, in the sense that an even stronger emphasis on openness and inclusiveness was found.

The evaluation study drawing upon the ART’s observations as well as the interviews moreover indicated an increase in the focus on and the use of AFR related concepts such as accountability, transparency, equity and publicity. There seemed to be a growing appreciation of these concepts as they assist the district administration in focusing the planning and allocation of resources towards fair resource allotment, enhanced management of the use of the financial resources as well as increased emphasis towards the vulnerable.

### Processes leading to transformation

The processes of transformation have according to the informants become more pronounced during the period that REACT was established and introduced the AFR conditions in the district. It would however be misleading to attribute this change solely to AFR, as there were several interventions taking place during the project period that were largely ‘pulling’ in a similar direction. Following the execution of the decentralisation policy in 1991, the MoH adopted ‘accountability’ and ‘quality leadership’ as guiding pillars for health sector reforms. These by now well established processes have articulated with those initiated by the ‘AFR based process’ in a manner that seems to have reinforced change. It should moreover be emphasized that during the project period, the district seemed to have had a leadership which embraced and championed the need for observing objectivity, equality, equity, transparency and inclusiveness. It is important to note that the District Medical Officer (DMO), who worked with the REACT team and highlighted these issues, remained the same throughout the entire project, a situation which permitted a consistency with regards to enforcement of the AFR concept over time in the district. It has been strongly documented that leadership qualities such as vision, alignment and building relationships are important in facilitating health care decision making [25].

The government’s emphasis on the growing number of stakeholders and committees to be involved in PS processes was said to help in galvanising fair PS in terms of enhanced budgeting and monitoring of resources. It was reported that there were changes with regards to both the number and type of stakeholders involved in PS processes. This more comprehensive composition of the DHMT has the potential of adding value to fair PS processes as new members are likely to bring with them different ways of addressing issues as well as principles deemed relevant, evidence and reasons that can guide stakeholders in developing priorities perceived as fair and relevant for the particular context. This seemed to strengthen the relevance condition of AFR which states that the rationales for a fair prioritization process must rest on the reasons that stakeholders can agree upon as relevant in the context, and rationales for PS decisions should aim at providing a reasonable explanation as to why certain rulings are made (13,15). Involving multiple stakeholders potentially helps to ensure that a wide range of relevant values and principles are taken into account and that mutual accountability between the health sector, its users and their communities is supported [26].

In addition to a broader platform from where to include values and principles in PS, broad participation in PS has the potential of increasing legitimacy of the PS and the likelihood of acceptance of priorities by the community. In addition, the contribution of stakeholders from outside the MoH creates potentials for tightening the checks and balances processes during the implementation process of priorities, a situation which is likely to result not only in improved PS processes, but ultimately in more efficient service delivery to the community.

The importance of the district motto *providing quality health services in partnership with the community* which was guiding health service provision within the district already at the onset of the project should be emphasized, as it was spelled out as a pillar for guiding planning processes. It has actually been acknowledged that institutional visions such as this motto can play a key role in promoting participation. International experience has shown that organizational contexts have the capacity to exert a strong enabling influence on public participation, the outcome of which will also be dependent on the existence of a participatory culture in a particular community [27,28].

Importantly, the motto emphasizes partnerships with community structures, and does not particularly focus on the committees at the district level which is important in this context. Apparently, much of the new focus on inclusiveness in PS processes was taking place at the DHMT level. The study indicated that although a positive change towards increased inclusiveness took place, new attempts by the DHMT at reaching the grassroots through ensuring that the community was represented in the meetings at district level remained challenging. This was primarily linked to the funding which created limitations with regard to transport, accommodation and food costs for community members attending PS meetings, but was also caused by people’s lack of interest and of awareness of the possibilities of participation, illiteracy as well as established top down PS processes starting from the MOH. There is a strong culture of endorsing the plans and principles made by the MoH and other higher level structures.

These severe financial, political and cultural limitations raise questions about the feasibility of ensuring inclusive PS processes. This scenario indeed may question the relevance of the principle of bottom up approaches to PS as outlined both in the decentralization policy for the MoH and the AFR framework. These questions resonate with the findings of other studies on PS decisions in developing countries, indicating that PS may be determined by guidelines from the central government; decisions are influenced by power and to a limited extent by the norms and values of the involved actors [4,5].

As we have seen, this problematic scenario does not mean however that the community structures have been completely ignored in the present attempts at securing stakeholder involvement in PS processes, and that they are incapable of changing. Efforts aimed at enhancing involvement of lower structures in PS are, as we have seen above, in place and are in the process of being strengthened through attempts at enhancing publicity and communication channels and processes. Staff and other members of the community were, according to all the study participants, to a larger extent than previously able to receive information on central PS processes through more outreach attempts. This apparent transition is located at the heart of AFR with its emphasis on PS decisions and their rationales being publicly accessible for fairness and justice to prevail [14]. It is vital that stakeholders are informed about decisions made in order to positively contribute towards such processes of setting priorities or to appeal against them [29,30]. Another example of the connection between district staff and the structures at lower levels was seen through the introduction of the Action Research Team (ART)’s ‘meeting points’ in the four zones of the district, which provided opportunities for health center and community representatives (Neighbourhood Health Committee members) to describe and evaluate priorities and suggest possible upgrades to the DHMT.

### Strengths and limitations

Although effort was made to include informants from many levels of decision making in the district, the study did not include experiences of community members. The study is thus limited to the perceptions provided by institutional stakeholders. Another weakness is that not all the content of the meetings between the district officials and the ART or the content of the other meetings attended by the ART was documented through recordings. The interviews in both the baseline and the evaluation study were however recorded and transcribed. The trustworthiness of the findings was moreover bolstered through the triangulation of data (IDIs, FGDs, minutes and observations from the ATR meetings and documentary reviews).

The possibility that people will talk particularly positively about a project, in this case the AFR intervention, to individuals who are coming to ‘evaluate’/assess ‘their own’ intervention furthermore cannot be ignored in discussing the credibility of the results, and we remained particularly sensitive to this potential bias during the analysis of the findings.

The structure and process of PS do not differ greatly between different districts in Zambia, and the local concepts and ideals which imply fairness are likely to be very similar across the country. Thus, we contend that central findings based on this study may provide an understanding of fairness in PS processes also elsewhere in Zambia. The description of constraints and challenges as well as the prevailing resources and potentials may moreover provide a vantage point for the exploration of enhancing fairness in priority setting processes also in other resource-constrained settings.

## Conclusion

The study has described the views of stakeholders on fairness and fair PS processes over a period of three years, the period in which AFR concepts were introduced in Kapiri-Mposhi District, Zambia. It has demonstrated that local concepts and ideals which suggested fairness are very much in line with the principles of liberal democratic thinking and were well established at time of the introduction of the AFR conditions. Gaps in terms of their application and the realization of fair PS processes were established both in the base line and in the evaluation study. The ART minutes, the periodic observations and the follow up evaluation study however revealed the strengthening of the knowledge of the concepts and practices of fair PS process during the project period. There seemed to be a stronger sense of assimilation and appreciation of these. Notable ideas that were given increased emphasis were transparency, accountability and publicity and a broadening of stakeholder involvement in PS processes. Several factors within the district during the project period were geared towards similar outcomes, and have most likely facilitated and enhanced the positive development of the AFR process. Most importantly, the leadership style at the time of the project period which advocated inclusiveness, broad consultation, objectivity and transparency in PS process contributed positively to fairness in PS. The current challenge for the DMHT and other planning structures in the district is to develop strategies of sustaining positive developments in PS processes and to ensure that they translate into improved health service delivery.

## Abbreviations

PS: Priority setting; AFR: Accountability for reasonableness; REACT: Response to Accountable priority setting for Trust in health systems; NGO: Non-governmental organization; MoH: Ministry of Health; DHMT: District Health Management Team; PHD: Provincial Health Director; NHSP: National Health Strategic Plan 2006-2011; FNDP: Fifth National Development Plan; MTEF: Medium Term Expenditure Framework; NHC: Neighbourhood Health Ccommittee; PHCS: Primary health care service; ART: Action Research Team; DMO: District Medical Officer; DEI: Describe-evaluate-improve; IDI: In depth interview; FGD: Focus group discussion.

## Competing interests

The authors declare that they have no competing interests.

## Authors’ contributions

All six authors contributed towards developing this manuscript. JMZ, CM and CMs carried out data collection and analysis. JMZ drafted the manuscript and CM, CMs, AKH, JB and AB contributed to the revising of this manuscript. All authors read and approved the final manuscript.

## Pre-publication history

The pre-publication history for this paper can be accessed here:

http://www.biomedcentral.com/1472-6963/14/75/prepub

## Supplementary Material

Additional file 1 Baseline data collection tools. Click here for file

Additional file 2 Specific instructions on final survey IDI. Click here for file
