# Supplementary material for: Increased fairness in priority setting processes within the health sector: the case of Kapiri-Mposhi District, Zambia
Source: BMC Health Serv Res. 2014 Feb 18;14:75. doi: 10.1186/1472-6963-14-75 (PMC3932790; doi:10.1186/1472-6963-14-75)
Supplement: Additional file 1 — Baseline data collection tools. [file 1472-6963-14-75-S1.pdf]

Baseline data collection tools

In depth interview guideline

Background information checklist

Date: ………………………
Interviewer: …………………..
Interviewee:
    Name: ………………………………………….
    Institution: …………………………………….
    Position: ………………………………………..
    Place of interview: …………………………….
    Age: ……………….
    Sex: ……………….
    Is the interviewee a leader involved in district planning? …………. Yes: No:

    If yes follow the questions for leaders. If no follow the questions for non-leaders.

Introduction

Introduce yourself
Introduce the research project.
Present all relevant permissions and introduction letters.
Inform the interviewee of the anonymity and confidentiality of the information provided
Inform the interviewee of the right to abstain from answering and from participating should he/she wish to do so
Ensure that if willing to participate he/she signs the consent form.

Bolded questions are suggested as main questions, to be ensured asked during the interview.

**Questions for leaders involved in district planning – follow questions in this columng**	**Questions for all others – follow questions in this column**
**Basic overview of priority setting processes**<br><br>**A) Process of setting priorities**<br><br>1. What is your understanding of priority setting?<br>2. What is the process of setting priorities?<br>**a. How are priorities set?**<br>b. What guidelines do you use in PS?<br>c. What are the stages followed in PS process?<br><br>**3. Who is involved in the PS process?**<br>a. How is the membership of those involved in PS process determined?<br>i. Probe: for age, gender, sector for consideration in determining membership for involvement in PS process<br>b. Who is consulted for inputs into the PS process?<br><br>4. When does the process start?<br>a. How long does it last?<br><br>5. Where does it take place?<br><br>6. Do you revisit the priorities set?<br>a. If yes or no why?<br><br>**B) Criteria used in PS process**<br><br>**7. What factors/criteria/values are taken into account when identifying priorities?**<br>8. Who decides on the factors to be taken into account?	**Basic overview of priority setting processes**<br><br>**A. Process of setting priorities**<br><br>2. What is your understanding of priority setting?<br>3. What is the process of setting priorities?<br>**a. How are priorities set?**<br><br>**4. Who is involved in the PS process?**<br>a. How is the membership of those involved in PS process determined?<br>i. Probe: for age, gender, sector for consideration in determining membership for involvement in PS process<br>b. Who is consulted for inputs into the PS process?<br><br>5. When does the process start?<br>a. How long does it last?<br><br>6. Where does it take place?<br><br>**A. Criteria used in PS process**<br><br>**7. What factors/criteria/values are taken into account when identifying priorities?**<br>8. Who decides on the factors to be taken into account?<br><br>**A. Evaluation**<br><br>**9. Who is involved in evaluating priority setting?**<br>10. Is this evaluation used in adjusting priorities?

**C) Evaluation**<br><br>**9. How do you evaluate your priority setting?**<br>10. Is this evaluation used in adjusting priorities?<br><br>**D) Implementation**<br><br>**11. Describe how your priorities are implemented**<br>12. Can you tell us the stages of implementing the priorities identified<br>13. What are the challenges of implementing the priorities identified?<br><br>Now tell the interviewee that we wish to ask for his/her understanding and opinions about the main concepts involved in the Accountability for Reasonableness framework.<br><br>**Fairness**<br><br>What is your understanding of fairness?<br>Do you think it is relevant in setting priorities? Explain<br>**Do you think the process you have described in A is fair? Explain**<br><br>**Accountability**<br><br>What is your understanding of accountability?<br>Do you think it is relevant in setting priorities? Explain<br>**Do you think the process you have described in A is accountable? Explain**<br><br>Specific question:<br><br>How do you disseminate the priorities set?<br>How do you disseminate the reasons for the priorities set?	**A. Implementation**<br><br>**11. Describe how priorities are implemented**<br><br>Now tell the interviewee that we wish to ask for his/her understanding and opinions about the main concepts involved in the Accountability for Reasonableness framework.<br><br>**Fairness**<br><br>What is your understanding of fairness?<br>Do you think it is relevant in setting priorities? Explain<br>**Do you think the process you have described in A is fair? Explain**<br><br>**Accountability**<br><br>What is your understanding of accountability?<br>Do you think it is relevant in setting priorities? Explain<br>**Do you think the process you have described in A is accountable? Explain**<br><br>Specific question:<br><br>How are priorities disseminatet?<br>How are reasons for priorities disseminatet?<br><br>**Publicity**<br><br>What is your understanding of publicity?<br>Do you think it is relevant in setting priorities? Explain<br>**Do you think the process you have described in A is publicly accessible? Explain**<br><br>Specific question:<br><br><u>Communication</u>

**Publicity**<br><br>What is your understanding of publicity?<br>Do you think it is relevant in setting priorities? Explain<br>**Do you think the process you have described in A is publicly accessible? Explain**<br><br>Specific question:<br><br><u>Communication</u><br><br>**How do you communicate with the recipients of the priorities?**<br><br>*If the interviewee is not a policy maker / involved in priority setting, he/she should explain*<br><br>How do you communicate with the policy makers and those setting priorities?<br><br><u>Transparency</u><br>Do you think the process of setting priorities described in A is transparent? Explain<br>How do you ensure that the process of priority setting is transparent?<br><br>*If the interviewee is not a policy maker / involved in priority setting, drop question (last question)*<br><br><br>**Relevance**<br><br>What is your understanding of relevance?<br>Do you think it is relevant in setting priorities? Explain<br>Do you think the process you have described in A takes relevance into account? Explain<br><br>Specific questions:<br><br><u>Information gathering</u><br>**What kind of information is presented for priority setting?**<br>*Probe on information on services, information on activities and information on resources*	**How is communication with the recipients of the priorities set conducted?**<br><br>How do you communicate with the policy makers and those setting priorities?<br><br><u>Transparency</u><br>Do you think the process of setting priorities described in A is transparent?<br><br><br>**Relevance**<br><br>What is your understanding of relevance?<br>Do you think it is relevant in setting priorities? Explain<br>Do you think the process you have described in A takes relevance into account? Explain<br><br>Specific questions:<br><br><br><u>Values and other criteria</u><br>**What values and other criteria do you consider important for priority setting?**<br>***Present a list of possible values if the question is not understood – human rights, equity, trust, quality, gender, priority to poor before rich, marginalized groups, etc.***<br><br>Do you think the priority setting process you have described in A is consistent with the values and other criteria you mentioned? Explain<br><br>**Appeal / Revision**<br><br>What is your understanding of appeal and revision?<br>Do you think it is relevant in setting priorities? Explain<br>**Do you think the process you have described in A includes appeal and revision mechanisms? Explain**<br><br>Specific questions:<br><br><u>Consensus</u><br>**How do you resolve disagreements with policy makers on a set priority?**

Who decides what sort of information is used for priority setting?
How is the information presented used for priority setting?

<u>Values and other criteria</u>
What values and other criteria do you consider important in your priority setting? *Present a list of possible values if the question is not understood – human rights, equity, trust, quality, gender, priority to poor before rich, marginalized groups, etc.*

Do you think the priority setting process you have described in A is consistent with the values and other criteria you mentioned? Explain

Appeal / Revision

What is your understanding of appeal and revision?
Do you think it is relevant in setting priorities? Explain
Do you think the process you have described in A includes appeal and revision mechanisms? Explain

Specific questions:

<u>Consensus</u>
How do you resolve disagreements on a priority?
Who is involved in resolving disagreements in priority setting?

Leadership and Enforcement

What is your understanding of leadership?
Do you think it is relevant in setting priorities? Explain
Are the leaders capable of implementing the priorities identified? Explain
Do you think the process you have described in A has the leadership needed to enforce Publicity, Relevance and Appeal? Explain

Who is involved in resolving disagreements in priority setting?

Leadership and Enforcement

What is your understanding of leadership?
Do you think it is relevant in setting priorities? Explain
Are the leaders capable of implementing the priorities identified? Explain
Do you think the process you have described in A has the leadership needed to enforce Publicity, Relevance and Appeal? Explain

Specific questions:

<u>Roles and Responsibilities</u>
How would you describe the transparency of roles and responsibilities in the priority setting process? (E.g. are they clear, are they known)
How would you describe the effectiveness of the roles and responsibilities in the priority setting process? (E.g. do leaders take responsibility and perform their roles)

<u>Skills</u>
How would you describe the skills of the leadership on
- decision making processes
- creating the inventory (services, activities and resources)
- resource shift and allocation?

If the interviewee is a leader:<br><br>How do you ensure enforcement and implementation of the priorities identified? Explain<br><br>Specific questions:<br><br><br>Roles and Responsibilities<br>How would you describe the transparency of roles and responsibilities in the priority setting process? (E.g. are they clear, are they known)<br>**How would you describe the effectiveness of the roles and responsibilities in the priority setting process? (E.g. do leaders take responsibility and perform their roles)**<br><br>Skills<br>How would you describe the skills of the leadership on<br>decision making processes<br>creating the inventory (services, activities and resources)<br>resource shift and allocation?	

End of interview

Thank the interviewee for their time and willingness to answer the questions and inform them of the future plans and intentions of the project.

Focus Group Discussion Guide

Background information checklist

Date
FGD facilitator
Referee
Place of interview
Participant group description
Participants (see table at end of guide)

Introduction
Introduce yourself
Introduce the research project.
Present all relevant permissions and introduction letters.
Inform the participants of the anonymity and confidentiality of the information provided
Inform the participants of the right to abstain from answering and from participating should he/she wish to do so
Ensure that if willing to participate he/she signs the consent form.

Basic overview of priority setting processes

E) Process of setting priorities

In reality, who is involved in the PS process?

a. How is the membership of those involved in PS process determined?
    i. Probe: for age, gender, sector for consideration in determining membership for involvement in PS process

F) Criteria used in PS process

What factors/values/criteria are taken into account when identifying priorities ? (record values)
Who decides on the factors to be taken into account?

G) Evaluation

How do you evaluate your priority setting and does it lead to change of priorities? (record meanings related to the 4 conditions)

H) Implementation

What are the challenges of implementing the priorities identified?

Now tell the interviewee that we wish to ask for his/her understanding and opinions about the main concepts involved in the Accountability for Reasonableness framework. Start with the general question on fairness:

Do you think that the priority setting process in your district is fair? (explain)

<u>Relevance</u>

Do you think the priority setting process in your district is relevant to your needs and challenges? Explain (Beware that this could lead to a long discussion on needs and leave less time on the priority setting process. Focus discussion to priority setting process)

<u>Specific questions</u>

Values and other criteria:
What values and other criteria do you consider important in your priority setting?
Present a list of possible values if the question is not understood – human rights, equity, trust, quality, gender, priority to poor before rich, marginalized groups, etc.

Do you think the priority setting process in your district takes on board the values and other criteria you mentioned? Explain

<u>Publicity</u>

Do you think the priority process and the reasons are widely made known and publiciced to the public and relevant stakeholders? E.g. communities, beneficiaries, patients, medical personnel, Explain

<u>Specific question</u>

Transparency:
Do you think the priority process in your district is transparent? To what extent and how is it or is it not transparent?

<u>Appeals / Revision</u>

Are you able to appeal and ask for revision if the priorities are not relevant to your values, needs and challenges? Note: If In-depth interviews reveal that there are no such mechanisms skip this question.

<u>Specific questions</u>

Consensus:
How do you resolve disagreements on a priority?

<u>Leadership and Enforcement</u>

Explain initiatives undertaken by leaders to ensure implementation of the priorities identified? (Probe for Publicity, Relevance and Appeal mechanism efforts)

Roles and Responsibilities:
How would you describe the transparency of roles and responsibilities in the priority setting process? (E.g. are they clear, are they known)
To what extend to do leaders understand their roles and implement their responsibility?

Skills:
How would you describe the skills of the leadership on resource shift and allocation? (How often, how, based on what information and criteria?)

<u>End of FGD</u>

Thank the interviewees for their time and willingness to answer the questions and inform them of the future plans and intentions of the project.

Name	Institution	Position	Age	Sex	Comments	Comments

Document and information source review checklist

Name of district
Name of registrar
Year

1) National health plans and policy guidelines (Especially look for mission, vision and values in 1-7 and whether they are maintained throughout the documents and in practice)
2) National resource allocation documents
3) Vertical programme document relevant for the district

4) District Plan
5) District Health Plans
6) District resource allocation documents
7) Distric Health Reports (Annua, Biannual, Quarterly)
8) DHMT management meeting minutes
9) District files, statutes and inventories – Establishement of DHMT (Job descriptions of leaders etc)
10) Planning documents of other programmes, donors and initiatives for use at district level (e.g. GTZ, USAID, DANIDA, IMCI, RCHS etc)
11) Minutes of relevant committees (E.g as Social Services Committees (Tanzania), District Health Boards,
12) Facility plans
13) Community plans
14) Media and information reports within the district (IEC, Press and Radio and TV reports) To be collected from the main stakeholders and the press.

Check documents for assessement of these topics:
Relevance

Reasons for priority setting

Publicity

Specifically look for
communication methods
transparency of the priority setting process

Specific documents: IEC, Media, Minutes, Reports

Leadership and enforcements

Specifically look for
transparency of roles and responsibilities
effectiveness of roles and responsibilities

Specific documents: Minutes, Job descriptions, District files and inventories

Networks of deliberation

Specifically look for
Who was involved in the priority setting process

Specific documents. Minutes, Reports, District Health Plans

Values and guiding principles

Specifically look for
What values and guiding principles were used to set priorities
What documents and guidelines were used to set priorities - including mission, and vision statements

Specific documents: District Health Plan, Minutes, Guidelines, Programme guidelines

Alignment to criteria

Specifically look for
Alignment procedures to align priorities to the values, guiding principles and guidelines used

Specific documents: District Health Plans, Minutes, Reports, Media

Resource allocation

Specifically look for
Resource patterns and allocation of resources
Shift of resources

Specific Documents: Plans, Resource allocation documents, Media

Policy pattern

Specifically look for
Descriptions of shift in policies and its reasons

Specific documents: Policy guidelines, Plans, Minutes, Reports, Media

End of document and information source guide

OBSERVATION GUIDE

The checklist should focus on activity, content, and results

INSTITUTIONAL DOMAIN TOOL

- Relevance
  - Is information gathered?
  - What values and criteria are discussed and used?
  - Description of background documents and the kind of information contained in the documents, quality and distribution
  - Other relevant information provided in the meeting

- Publicity
  - Does the meeting have a strategy or discuss on how to communicate and to get transparency (what are the activities, contents and results)
  - Is an agenda for the meeting communicated to the participants in advance?
  - Is there any communication regarding the occurrence of the meeting?

- Appeals and revision
  - Does the process discuss the appeal process
  - Describe the process and what is your opinion
  - Are channels of appeal discussed and agreed upon?
  - How was consensus reached?
  - Are decisions challenged and revised? How?

- Leadership

  (Are the AFR conditions taken into account when discussions on PS – for analysis section)
  - Is the leadership in control of the meeting (strict agenda, conducting the meeting and reaching consensus)
  - Are the relevant materials made available
  - Are there any consequences
  - Are the individuals held accountable for their actions of omission or commission in discharging their responsibilities/tasks
  - Are there any shifts in policy? What caused the shift?
  -

PUBLIC DOMAIN TOOL

- Relevance

  - Are the any expressions of consent or dissent about the priorities communicated to the public?
  - How do the disseminators of information handle the dissent?

- Publicity

    - Is there any information from the meeting reaching the public? Describe the content of the information
    - Does the public know about the meeting? Public awareness to the meeting, e.g. posters, radio announcements

- Appeal/revision

    - Were the channels of appeal/revision communicated to the public? How? (Probe for mechanisms of communication)

- Leadership

    - Is there competence in communicating to the public?
    - Are the leaders held accountable for their actions of omission or commission in discharging their responsibilities/tasks
    - Are there any shifts in policy focus? What caused the shift?
