# Supplementary material for: Increased fairness in priority setting processes within the health sector: the case of Kapiri-Mposhi District, Zambia
Source: BMC Health Serv Res. 2014 Feb 18;14:75. doi: 10.1186/1472-6963-14-75 (PMC3932790; doi:10.1186/1472-6963-14-75)
Supplement: Additional file 2 — Specific instructions on final survey IDI. [file 1472-6963-14-75-S2.pdf]

Specific instructions on final survey IDI

List of possible interviewees:

List of people / positions to be interviewed in the final survey.
DHMT core members – 4 interviewees and must include the Administrator and the DMO
Disease specific focal persons (HIV, Malaria, Reproductive Health) – 3 interviewees
District Health Management Boards – 2 interviewees
District administrators – (e.g. District Executive Director (DED), District Planning Officer (DPLO), District Human Resource Officer (DHRO) 2-3 interviewees
Health facility in charge – 2 interviewees
Leaders of prominent NGOs in district (one of them not being a Voluntary Agency health service provider) – up to at most three interviewees
Health workers at the district hospital – up to two knowledgeable persons from hospital – including superintendant if he/she has been involved.

Total Number up to 19.

Criteria for inclusion of interviewees: work experience in the current position for at least one year. If the suggested person does not qualify according to these criteria find another suitable person.

Decisions on who to interview among a group of possible interviewees are taken by the research team in collaboration with the country coordinator and scientific committee.

FINAL SURVEY TOOL - DRAFT

**A) PROCESSES OF DECISION MAKING**
1. How is management of health organized in the district?<br>What has changed in the last three years?, What has led to these changes?<br>***Probe***: are there other factors that have caused changes?
2. How do you deal with problems that emerge in the process of running the activities<br>What has changed in the last three years?, What has led to these changes?<br>***Probe***: are there other factors that have caused changes?
**B) PROCESSES OF SETTING PRIORITIES**
3. What is your understanding of priority setting?
4. What is the process of setting health priorities (e.g. stages in the process, length of the process, guidelines)?
5. Who is involved in the health sector priority setting process?<br>Probe for the influence of ________ (e.g. gender, economic status, organizations, formal educational level, age, ethnicity, religious affiliation).<br>Have there been any changes in number and type of people involved in the last three years? ***Probe***: how and why.
**C) CRITERIA USED IN PRIORITY SETTING PROCESSES**
6. What criteria are taken into account when identifying priorities?<br>What has changed in the last three years?, What has led to these changes?<br>***Probe***: are there other factors that have caused changes?
7. What criteria do you consider for allocating resources in the district?<br>What has changed in the last three years?, What has led to these changes?<br>***Probe***: are there other factors that have caused changes?
**D) EVALUATION**
8. How do you evaluate your priority setting?<br>What has changed in the last three years?, What has led to these changes?<br>***Probe***: are there other factors that have caused changes?
9. Is this evaluation used in adjusting priorities?
**E) IMPLEMENTATION AND MANAGEMENT**
10. How are your health priorities implemented<br>What has changed in the last three years?, What has led to these changes?<br>***Probe***: are there other factors that have caused changes
11a. How is human resource management handled by the district management team ? Probe on who participates, what guidelines are in place, fairness in distribution, training, what appeal mechanisms exist for a health worker.<br>11b. What happens if a decision to be made would go against the guidelines?
12. Have decisions made in the last three years improved trust between management and staff ? ***Probe***: how
13. How did you implement the REACT Project?<br>***Probe***: for challenges faced

Note to the interviewer: tell the interviewee that we wish to ask for his/her understanding and opinions about the main concepts involved in the AFR framework. Important to get explanation from the interviewee on the following sections.
**F) FAIRNESS**
14. What is your understanding of fairness? Probe on its importance in setting priorities<br>What has changed in the last three years?, What has led to these changes?<br>***Probe***: are there other factors that have caused changes?
**G) ACCOUNTABILITY**
15. What is your understanding of accountability? Probe on its importance in setting priorities<br>What has changed in the last three years?, What has led to these changes ?<br>***Probe***: are there other factors that have caused changes
**H) RELEVANCE**
16a. What is the importance of relevance in setting priorities ?<br>What has changed in the last three years?, What has led to these changes ?<br>***Probe***: are there other factors that have caused changes<br>16b. What criteria do you consider important in your priority setting?
**I) PUBLICITY**
17. What is your understanding of publicity? Probe on its importance in setting priorities<br>What has changed in the last three years?, What has led to these changes?<br>***Probe***: are there other factors that have caused changes<br>***Probe*** on dissemination, communication and transparency
**J) APPEAL / REVISION**
18a. What is your understanding of appeal and revision? Probe on its importance in setting priorities<br>What has changed in the last three years?, What has led to these changes?<br>***Probe***: are there other factors that have caused changes<br>18b. Have there been any appeals in the last three years. If yes, how have these been handled ? If no, does the district have any appeal mechanisms ?
**K) LEADERSHIP / ENFORCEMENT OF AFR**
19a. What is the importance of leadership in enforcing the AFR conditions ?<br>What has changed in the last three years?, What has led to these changes?<br>***Probe***: are there other factors that have caused changes<br>19b. What measures have been put in place in the last three years to enforce the AFR conditions (relevance, publicity, appeal)?
**L) CONCLUDING QUESTION**
20. Is there anything you would like to add or comment on AFR ?
